# THE IMPACT OF CONTRACT NURSE UTILIZATION ON NURSING HOME QUALITY

**DOI:** 10.1093/geroni/igad104.0746

**Published:** 2023-12-21

**Authors:** Ganisher Davlyatov, Robert Weech-Maldonado, Rohit Pradhan, Akbar Ghiasi, Justin Lord

**Affiliations:** University of Oklahoma Health Sciences Center, Oklahoma City, Oklahoma, United States; University of Alabama at Birmingham, Birmingham, Alabama, United States; Texas State University, San Marcos, Texas, United States; University of the Incarnate Word, San Antonio, Texas, United States; Louisiana State University at Shreveport, Shreveport, Louisiana, United States

## Abstract

Nurse staffing intensity and skill mix have a significant impact on NH quality of care. For instance, higher staffing levels, particularly for registered nurses (RNs), have been associated with better resident outcomes. NHs have increasingly relied on contract nurse staffing to address pressing and persistent nursing shortages. Research suggests that contract nurses may be unfamiliar with institutional policies and procedures or organizational culture. They may require increased supervision, be less participatory in teamwork, and may increase the workload for the permanent staff. The objective of this study is to examine the association between contract nurse utilization and quality of care at US nursing homes.We used secondary data from PBJ nurse staffing, Nursing Home Compare, Healthcare Cost Report Information System (HCRIS) Dataset, and the American Community Survey (ACS) for the period 2017-2021. The dependent variable was CMS quality star rating, and the independent variables were contract nurse to total nurse proportions for each of RNs, LPNs, and CNAs. We employed multivariate random effects ordered logistic regression to test the hypothesis. We found that across all our three models (RNs, LPNs, CNAs), quality star ratings were negatively associated with contract nurse utilization. A 10% increase in contract nurse proportion was associated with a 10% decrease in the odds of reporting a higher quality star rating (OR=0.99, p<0.001). Our results suggest the increased utilization of contract nurse staffing may have a negative impact on US NH quality---an important issue that has agitated policymakers for nearly three decades.

